# Spider Venom Peptides as Potential Allosteric Inhibitors of Undecaprenyl Diphosphatase (UppP) from *Acinetobacter baumannii*: In Silico Identification and Structural Analysis

**DOI:** 10.3390/toxins18050210

**Published:** 2026-04-30

**Authors:** Yamil Liscano, Juan M. Álvarez-Caballero, Alberto Aragón-Muriel

**Affiliations:** 1 Grupo de Investigación en Salud Integral (GISI), Facultad de Salud, Universidad Santiago de Cali, Cali 760035, Colombia; 2 Grupo de Quimica y Bioprospeccion de Productos Naturales (QUIBIP), Departamento de Química, Universidad del Magdalena, Santa Marta 470004, Colombia; jalvarez@unimagdalena.edu.co

**Keywords:** *Acinetobacter baumannii*, Undecaprenyl Diphosphatase (UppP), spider venom, molecular docking, antimicrobial resistance, antimicrobial peptides (AMPs), allosteric inhibitors

## Abstract

The antimicrobial resistance of *Acinetobacter baumannii* necessitates the development of novel therapeutic strategies targeting essential enzymes such as Undecaprenyl Pyrophosphate Phosphatase (UppP). This study explored spider venom peptides in silico as potential allosteric inhibitors of *A. baumannii* UppP. A systematic literature review was conducted to select eight α-helical peptides with reported anti-*A. baumannii* activity, followed by their computational physicochemical characterization. Three-dimensional models of *A. baumannii* UppP and the candidate peptides were generated, and a putative allosteric binding site was validated through molecular docking of a known inhibitor of the BacA homolog. The eight peptides were subsequently docked to this validated site using HADDOCK. Results revealed variable binding affinities; peptides LC-AMP-I1, Lycosin-II, and GK37 exhibited the most favorable HADDOCK scores and extensive interaction networks, consistent with their reported high antimicrobial potency. Other candidates, notably Lt-MAP2, showed low binding affinity but high predicted synergistic potential. These findings identify promising spider venom peptide candidates, suggesting dual (membrane disruption/UppP inhibition) or synergistic mechanisms of action, and validate UppP as a viable pharmacological target for peptide-based inhibitors.

## 1. Introduction

Antimicrobial resistance represents one of the most pressing threats to global public health, with *Acinetobacter baumannii* standing as a paradigmatic example of this crisis. Classified by the World Health Organization as a critical priority pathogen, this Gram-negative coccobacillus is responsible for severe nosocomial infections and has demonstrated a remarkable capacity to acquire multidrug-resistant (MDR), extensively drug-resistant (XDR), and pandrug-resistant (PDR) phenotypes through diverse mechanisms, including efflux pump overexpression, inactivating enzyme production, and target mutations, that have drastically depleted available therapeutic options [1,2,3,4,5].

Among underexplored antibacterial targets, Undecaprenyl Pyrophosphate Phosphatase (UppP), a conserved transmembrane enzyme essential for recycling the lipid carrier C55-P and sustaining peptidoglycan biosynthesis, has emerged as a compelling candidate. Its inhibition collapses cell wall synthesis and leads to bacterial lysis, as demonstrated by bacitracin and validated pharmacologically for the homologous BacA enzyme by Jukič et al. [6].

Beyond *A. baumannii*, UppP is a conserved and physiologically essential enzyme across diverse bacterial lineages. In *Escherichia coli*, UppP (originally named BacA) accounts for approximately 75% of the C55-PP phosphatase activity, with residual activity distributed among the PAP2-family enzymes PgpB, YbjG, and YeiU [7]. In *Bacillus subtilis*, UppP and its paralog BcrC constitute a synthetic–lethal gene pair whose combined activity is essential for peptidoglycan and wall teichoic acid biosynthesis; their depletion severely impairs growth, morphology, and sporulation [8,9]. Similar essentiality has been documented in Gram-positive pathogens such as *Streptococcus mutans* [10] and *Enterococcus faecalis* [11], where UppP activity additionally modulates intrinsic bacitracin susceptibility. In *Helicobacter pylori*, the UppP ortholog HupA is required for gastric colonization [12]. The recent resolution of the crystal structures of UppP from *E. coli* [13] and of its BacA homolog [14] has further clarified the mechanistic and structural basis of this enzyme family, highlighting conserved catalytic motifs that remain largely unexploited as pharmacological targets.

Despite its essentiality, UppP remains a pharmacologically underexploited target. The classical cyclic peptide antibiotic bacitracin, clinically used against Gram-positive infections, indirectly disrupts UppP function by sequestering its substrate C55-PP through a Mg^2+^- or Zn^2+^-dependent 1:1 complex, thereby preventing lipid carrier recycling and triggering cell lysis [15,16]. However, bacitracin neither targets the enzyme directly nor displays meaningful activity against Gram-negative pathogens such as *A. baumannii*, and widespread resistance mechanisms, including upregulation of UppP itself and of BcrAB-type ABC transporters, have limited its clinical utility [17]. More recently, Jukič et al. (2022) [6] reported the first small-molecule direct UppP/BacA inhibitors through virtual high-throughput screening, validating the enzyme as a druggable target and identifying an allosteric pocket distinct from the catalytic site. In parallel, rationally redesigned bacitracin analogs active against vancomycin-resistant pathogens have been described [18], underscoring a renewed interest in UppP-centered chemotherapy. In this context, no peptide-based inhibitors of *A. baumannii* UppP have been reported, creating a clear opportunity to explore alternative chemical classes. Among these, natural product libraries, particularly those derived from venomous organisms, offer a highly specialized chemical space of bioactive molecules that have evolved for precise molecular recognition at the membrane interface [19].

Spider venom antimicrobial peptides (AMPs), which are typically cationic, amphipathic, and α-helical molecules optimized for interaction with lipid environments, represent a promising class of agents to target such transmembrane proteins [20,21], particularly given the intrinsic difficulty of developing resistance against membrane-active mechanisms. Several spider-derived AMPs have already demonstrated potent and diverse antibacterial mechanisms that justify their exploration as therapeutic leads. Lycosin-I, isolated from *Lycosa singoriensis*, exhibits rapid and broad-spectrum bactericidal activity at low micromolar concentrations, acts synergistically with conventional antibiotics, and permeabilizes the bacterial membrane, as evidenced by SYTOX uptake assays and scanning electron microscopy [22]. Its homolog Lycosin-II retains potent activity (MIC = 3.1–6.3 µM) against MDR clinical isolates of *A. baumannii*, with Mg^2+^-competition experiments confirming direct interaction with the negatively charged bacterial surface [23]. Beyond classical wolf spiders, Lycotoxin-Pa4a from *Pardosa astrigera* was recently shown to simultaneously disrupt outer and cytoplasmic membranes of both Gram-negative and Gram-positive bacteria while exerting anti-inflammatory effects [24], and gomesin from *Acanthoscurria gomesiana* displays potent activity against Gram-negative bacteria and has been rationally redesigned to enhance its therapeutic index [25]. These examples illustrate that spider-derived AMPs combine rapid membrane-active killing with potential for mechanistic diversification, making them particularly attractive candidates for combating MDR pathogens. However, their specific interactions with UppP remain uncharacterized. Here, we report an integrated computational framework, combining systematic literature review, physicochemical characterization, and molecular docking, to identify and evaluate eight spider venom peptides as potential allosteric inhibitors of *A. baumannii* UppP, defining their binding modes and prioritizing candidates for experimental validation.

## 2. Results

### 2.1. Peptide Selection via Systematic Literature Review

**Figure 1:**
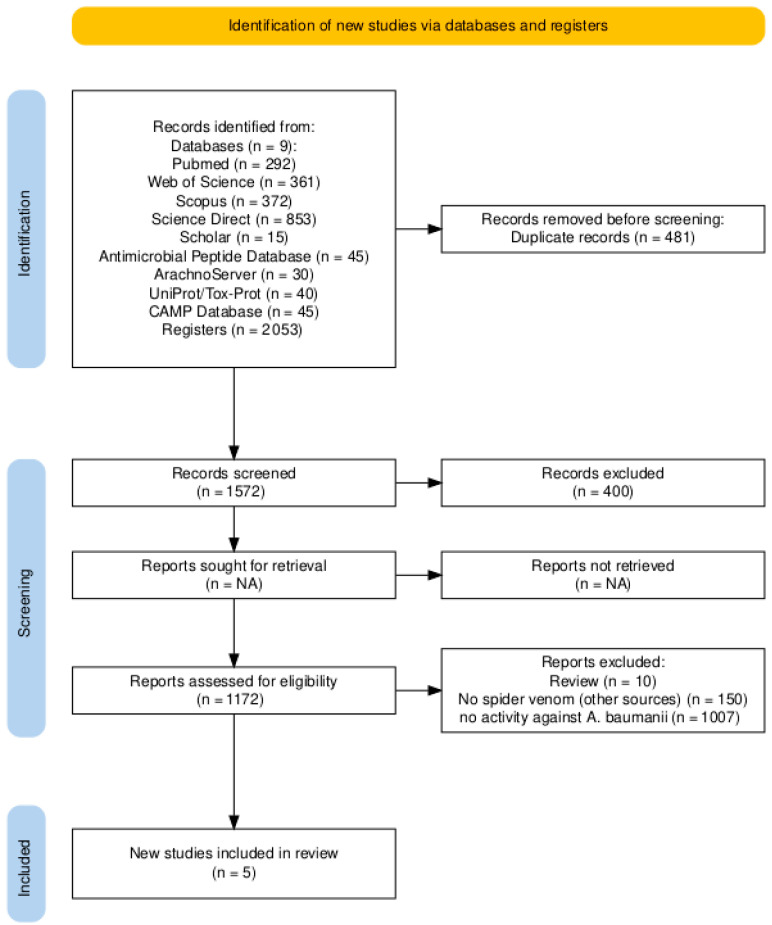
Flow diagram detailing the process of identification, screening, eligibility, and inclusion of studies. The methodology follows PRISMA (Preferred Reporting Items for Systematic reviews and Meta-Analyses) guidelines. Abbreviations: CAMP, Collection of Anti-Microbial Peptides; UniProt/Tox-Prot, UniProt Toxin and Toxin Target Database.

The initial systematic search identified a total of 2053 potentially relevant articles. After removing 481 duplicate records, 1572 unique articles were screened based on title and abstract, of which 400 were excluded for not meeting initial thematic criteria. Subsequently, the eligibility of 1172 full-text reports was assessed. In this phase, 1167 studies were excluded for various reasons: 10 were reviews, 150 described non-spider venom peptides, and 1007 did not report specific activity against *A. baumannii*. This selection process culminated in the inclusion of 5 studies meeting all predefined criteria. The detailed selection workflow is presented in Figure 1.

### 2.2. Characterization of Selected Spider Venom Peptides

From the five included studies, information on eight peptides with demonstrated activity against *A. baumannii* was extracted (Table 1). This candidate set represents a diverse cohort, including native sequences isolated from venom (e.g., LC-AMP-I1 from *Lycosa coelestis* and GK37 from *Oxyopes forcipiformis*) and synthetic analogs (e.g., Lt-MAP2 and Lt-MAP3) derived from parental peptides of *Lachesana tarabaevi*. Reported potency, measured as the Minimum Inhibitory Concentration (MIC), varies significantly, from high potency (MIC = 3.1–6.3 µM for Lycosin-II) to considerably weaker activity (MIC = 128 µg/mL for Lt-MAP3). Despite this functional and origin diversity, a unifying structural feature is that all peptides are reported as α-helical and mostly amphipathic, suggesting this conformation is fundamental to their antibacterial mechanism.

### 2.3. Physicochemical Characterization and In Silico Activity Prediction

Physicochemical properties were evaluated to elucidate molecular features influencing antimicrobial activity (Table 2). Analysis confirmed all candidates are cationic, with net charges ranging from +4 to +8. The peptides showed notable diversity: length varied from 13 residues (Latarcin analogs) to 40 residues (GK37). Parameters for membrane interaction, such as hydrophobicity (H) and hydrophobic moment (μH), showed significant divergence. For instance, Lt-MAP2 exhibited the highest hydrophobic moment (μH = 0.839), indicating pronounced facial amphipathicity, while Lycosin-II presented a much lower value (μH = 0.26). All peptides were predicted as antimicrobial by DBAASP. Predictions of synergy with conventional antibiotics revealed varied profiles, suggesting complementary mechanisms of action.

### 2.4. Comparative Analysis of Activity and Physicochemical Properties

**Figure 2:**
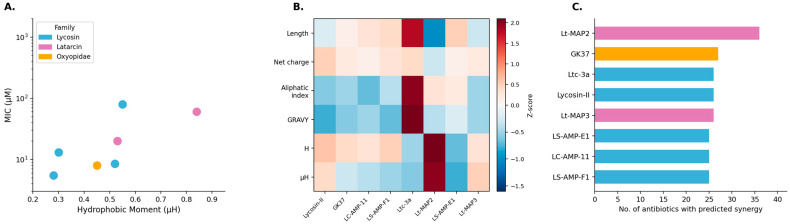
Comparative visual analysis of the selected peptides. (**A**) Correlation between hydrophobic moment (µH) and minimum inhibitory concentration (MIC) on a logarithmic scale. Data points are color-coded according to peptide family. (**B**) Heatmap showing normalized values (Z-scores) of the main physicochemical properties for each peptide. (**C**) Bar chart classifying peptides according to the total number of antibiotics with which a synergistic interaction is predicted. Abbreviations: MIC, Minimum Inhibitory Concentration; µH, Hydrophobic Moment.

To integrate the reported potency data (Table 1) with the physicochemical profiles (Table 2), a comparative visual analysis was performed (Figure 2). The scatter plot (Figure 2A) reveals a complex, non-linear relationship between amphipathicity (μH) and antimicrobial potency (MIC). Contrary to intuitive expectations, maximum potency (i.e., the lowest MIC) does not correlate with the highest μH values. Instead, highly potent peptides such as Lycosin-II, LS-AMP-F1, and GK37 cluster within an optimal μH range (approximately 0.25–0.40). Regarding the candidate Lt-MAP2, while some literature reports indicate an MIC of 8 µg/mL [26], the comparative analysis against the most potent leads in this dataset suggests a relatively lower intrinsic potency (MIC > 100 µM) in terms of molar concentration. This indicates that the peptides with the highest amphipathicity in the dataset, Lt-MAP3 (μH ≈ 0.70) and Lt-MAP2 (μH ≈ 0.84), are precisely those exhibiting the weakest individual bactericidal activity compared to the Lycosin family.

The heatmap (Figure 2B) provides a more granular view of these differences by utilizing Z-score normalization. This statistical transformation standardizes the physicochemical variables to a common scale (mean 0, standard deviation 1), enabling the identification of specific physicochemical strategies employed by each peptide family regardless of their original measurement units. This visualization highlights these distinct profiles, demonstrating that peptides employ different physicochemical strategies. For instance, GK37 appears as an outlier due to its greater length and high net charge (darker tones), whereas the Latarcin analogs (Lt-MAP2/3) stand out for their elevated aliphatic index and hydrophobic moment.

The most significant insight emerges when contrasting intrinsic potency (Figure 2A) with predicted synergistic potential (Figure 2C). An apparent paradox is identified: Lt-MAP2, the peptide with the comparatively lower individual antimicrobial activity, is predicted to be the most promising synergistic agent, with the potential to interact with more than 35 antibiotics. This finding underscores the importance of not discarding candidates based solely on intrinsic potency. The high amphipathicity of Lt-MAP2 suggests a specialized role; while it may not be sufficient to exert lethal activity on its own at low concentrations, it is likely ideally suited for a membrane-permeabilization mechanism. By destabilizing the lipid bilayer without necessarily causing immediate lysis, such peptides can facilitate the entry of other drugs into the cytoplasm, thereby potentiating the effect of conventional antibiotics that hit intracellular targets [19].

Given the diverse physicochemical profiles and the hypothesized mechanisms of action (direct activity vs. synergy) suggested by this analysis, structural evaluation through molecular docking of the eight candidate peptides was warranted to investigate their interactions with UppP.

### 2.5. 3D Modeling and Binding Site Validation Using a Control Ligand

To proceed with the structural analysis, high-fidelity three-dimensional models of the receptor and all ligands were generated. Since no experimental structure exists for UppP from *A. baumannii* (strain ACICU), a model was generated using AlphaFold2. The resulting model proved to be of high quality, with an average pLDDT score above 90 and a ProSA-web Z-score of −7.9, indicating a topology and energetic quality comparable to those of experimentally resolved structures.

**Figure 3:**
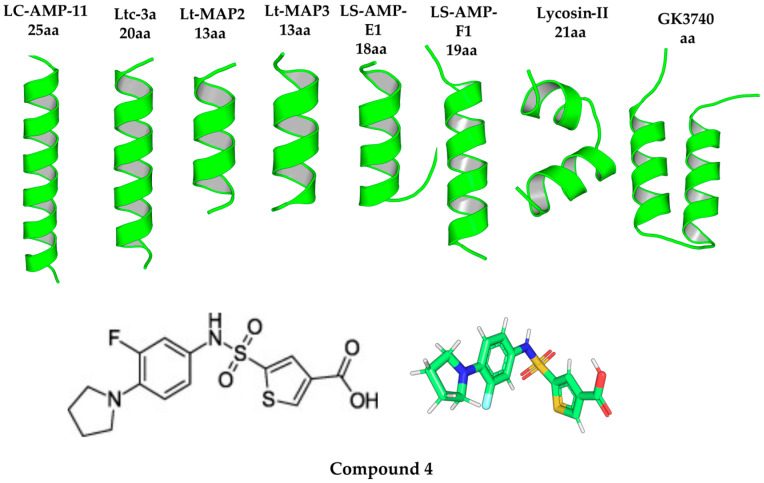
Structures of the analyzed ligands. (**Top**) 3D models of the eight selected spider peptides. (**Bottom**) 2D structure and 3D model of the control ligand, 5-((3-fluoro-4-(pyrrolidin-1-yl)phenyl)sulfonamido)thiophene-2-carboxylic acid. In the 3D model, atoms are represented by the following colors: carbon (green), oxygen (red), nitrogen (blue), sulfur (yellow), fluorine (cyan), and hydrogen (white).

In parallel, the 3D structures of the eight candidate peptides were modeled using PEP-FOLD, confirming that all adopt the α-helical conformation reported in the literature (Figure 3, Top). As a control ligand, Compound **4** was selected—an inhibitor of the homologous enzyme BacA reported by Jukič et al. [6]. Its 2D and 3D structures are shown in Figure 3 (Bottom).

**Figure 4:**
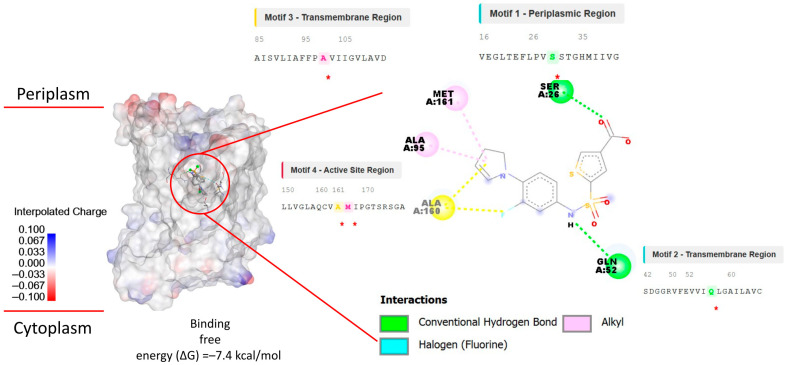
Analysis of the binding mode of the control ligand at the active site of *A. baumannii* UppP. (**Left**) Three-dimensional view of the docked complex. The control ligand (shown as sticks) is depicted bound to a hydrophobic pocket in the transmembrane region of UppP. The protein surface is colored according to electrostatic potential (blue: positive charge, red: negative charge). (**Right**) 2D diagram of ligand–protein interactions. Red stars in the sequence insets highlight the specific amino acids within the conserved motifs that are involved in the binding. A conventional hydrogen bond (green dashed line) is observed with residue Gln52 (Motif 2). Multiple hydrophobic interactions (Alkyl, pink dashed lines) and Pi-Alkyl interactions (yellow dashed lines) are predicted with residues Ser26 (Motif 1), Ala95 (Motif 3), Ala160, and Met161 (Motif 4). Additionally, a halogen (fluorine) interaction (cyan dashed line) is formed between the ligand and the receptor. The insets map these key residues to their respective conserved motifs within the UppP sequence.

Molecular docking of this control ligand against our UppP model was performed to validate a plausible binding site (Figure 4). Analysis of the docking revealed a stable, high-affinity binding mode within an allosteric pocket located in the transmembrane region of the protein (Figure 4, Left). This binding mode is stabilized by key interactions: a conventional hydrogen bond between the sulfonamide group of the ligand and residue Gln52 (Motif 2), and a network of hydrophobic contacts (Alkyl and Pi-Alkyl) with residues from other conserved motifs, including Ser26 (Motif 1), Ala95 (Motif 3), and Ala160/Met161 (Motif 4) (Figure 4, Right).

It is essential to note that this validated allosteric pocket differs from the catalytic site (involving Glu17 and Arg174) described for the homologous BacA. Having validated this allosteric binding site as a viable pharmacological target, it was used as the target region for the subsequent virtual screening of the eight candidate spider peptides.

### 2.6. Molecular Docking and Interaction Analysis of Spider Venom Peptides at the Allosteric Site of UppP

**Figure 5:**
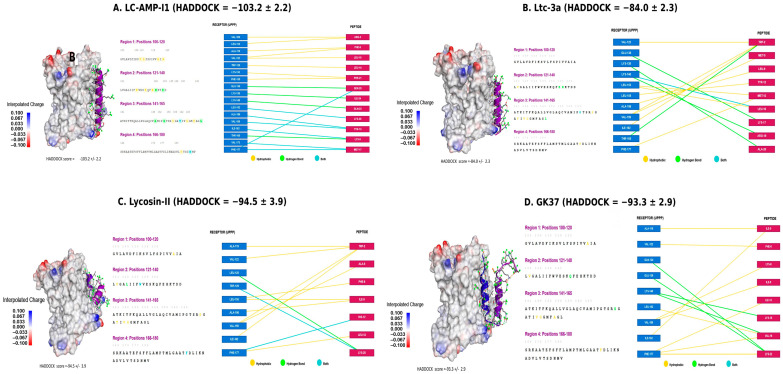
Molecular docking analysis of the four highest-affinity spider venom peptides at the allosteric site of *A. baumannii* UppP. Each panel (**A**–**D**) displays a three-dimensional representation of the docked complex (**left**) and a residue-level 2D interaction map (**right**). The UppP receptor surface is colored by interpolated electrostatic potential (blue: positive; red: negative); the peptide is shown as a purple α-helix. Interaction types are color-coded based on the legend: hydrophobic contacts (yellow), hydrogen bonds (green), and combined interactions (cyan). Note: combined interactions are not observed in panel D. HADDOCK scores are as follows: (**A**) LC-AMP-I1 (HADDOCK = −103.2 ± 2.2); (**B**) Ltc-3a (HADDOCK = −84.0 ± 2.3); (**C**) Lycosin-II (HADDOCK = −94.5 ± 3.9); (**D**) GK37 (HADDOCK = −93.3 ± 2.9). The docking results of the remaining four peptides (Lt-MAP2, Lt-MAP3, LS-AMP-E1, and LS-AMP-F1) are provided in the Appendix A (Figure A1).

**Figure A1:**
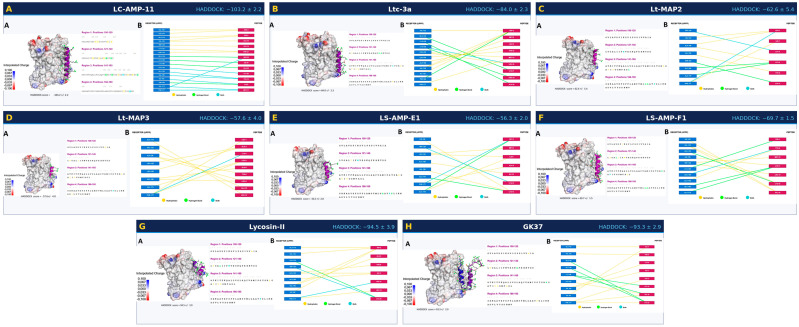
Molecular docking analysis of eight spider venom peptides at the allosteric site of *A. baumannii* UppP. Each panel (**A**–**H**) corresponds to one peptide candidate and displays two components: a three-dimensional representation of the docked complex (**left**), in which the UppP receptor surface is colored according to interpolated electrostatic potential (blue: positive charge; red: negative charge) and the peptide is shown as a purple alpha helix bound within the transmembrane allosteric groove; and a residue-level 2D interaction map (**right**), detailing the specific contacts established between UppP residues (left column) and peptide residues (right column) across four conserved receptor regions (Regions 1–4, covering positions 100–180). The sequence insets within the 2D map explicitly define these four conserved motifs, with specific residues and position numbers highlighted with specific colors corresponding to each region (Yellow for Region 1, Pink for Region 2, Green for Region 3, Cyan for Region 4). Interaction types are color-coded based on the internal legend: hydrophobic contacts (yellow lines), hydrogen bonds (green lines), and combined interactions (blue lines). (Note: hydrogen bonds are not present in panel (**D**), and combined interactions are not present in panel (**H**)). HADDOCK scores are indicated in the header of each panel: (**A**) LC-AMP-I1 (HADDOCK = −103.2 ± 2.2); (**B**) Ltc-3a (−84.0 ± 2.3); (**C**) Lt-MAP2 (−62.6 ± 5.4); (**D**) Lt-MAP3 (−57.6 ± 4.0); (**E**) LS-AMP-E1 (−56.3 ± 2.0); (**F**) LS-AMP-F1 (−69.7 ± 1.5); (**G**) Lycosin-II (−94.5 ± 3.9); (**H**) GK37 (−93.3 ± 2.9).

The results of the virtual screening of all eight spider venom peptide candidates against the validated allosteric site of UppP are presented in Figure 5 and Figure A1. Across the cohort, HADDOCK scores ranged from −56.3 ± 2.0 to −103.2 ± 2.2, and the interaction profiles revealed three distinct mechanistic categories that are discussed below.

The most exceptional docking profile was obtained for LC-AMP-I1 (Figure 5A), with a HADDOCK score of −103.2 ± 2.2, the most favorable value in the entire series, suggesting the formation of the most stable complex with the allosteric site of UppP. Crucially, this outstanding in silico affinity directly correlates with its potent biological activity (MIC = 5–10 µM), one of the lowest in the group. The 3D representation demonstrates remarkable shape and charge complementarity between the peptide helix and the electrostatic groove of the receptor, while the 2D interaction map reveals an extraordinarily dense and diverse contact network involving more than ten peptide residues, anchored through a combination of multiple hydrophobic interactions (yellow lines), specific hydrogen bonds (green lines), and mixed interactions (cyan lines). LC-AMP-I1 thus emerges as a unique candidate combining potent intrinsic antimicrobial activity with the highest predicted affinity for the allosteric target, suggesting a potential dual mechanism of action: membrane disruption and simultaneous specific enzymatic blockade.

Two additional peptides, Lycosin-II (Figure 5C; HADDOCK = −94.5 ± 3.9) and GK37 (Figure 5D; HADDOCK = −93.3 ± 2.9), also exhibited strong in silico binding, ranking second and third in the cohort, respectively. In both cases, the high predicted affinity aligns closely with their potent biological activities (Lycosin-II: MIC = 3.1–6.3 µM; GK37: MIC = 6.209 µM). The interaction map of Lycosin-II (Figure 5C) shows a diversified and robust binding mode stabilized by a well-distributed network of hydrophobic contacts, hydrogen bonds, and mixed interactions, crucial for anchoring within the transmembrane environment. GK37, which is structurally unique in the cohort due to its considerable length (40 residues) and high net charge (+8), engages the allosteric groove through a broad set of hydrophobic and hydrogen bond contacts spanning multiple receptor residues (Figure 5D). The congruence between in vitro potency and in silico affinity positions both peptides, alongside LC-AMP-I1, as strong candidates for a dual mechanism of action involving both membrane disruption and allosteric UppP inhibition.

A second category is represented by Ltc-3a (Figure 5B; HADDOCK = −84.0 ± 2.3) and LS-AMP-F1 (Figure A1D; HADDOCK = −69.7 ± 1.5), which display moderate-to-strong binding affinities combined with notably high biological potencies (Ltc-3a: MIC = 4 µg/mL; LS-AMP-F1: MIC = 3.1–25 µM). In both cases, the interaction networks show good distributions of hydrophobic contacts, hydrogen bonds, and mixed interactions, though qualitatively less extensive than those of the top-ranking candidates. This contrast between excellent in vitro potency and a strong but not maximal in silico affinity suggests that the primary mechanism of action for these peptides may be more inclined toward membrane disruption, with allosteric UppP engagement representing a secondary or additive contribution to their overall lethality.

The remaining three candidates, Lt-MAP2 (Figure A1A; HADDOCK = −62.6 ± 5.4), Lt-MAP3 (Figure A1B; HADDOCK = −57.6 ± 4.0), and LS-AMP-E1 (Figure A1C; HADDOCK = −56.3 ± 2.0), exhibited the weakest binding affinities in the cohort, consistent with their comparatively lower intrinsic antimicrobial activities (MIC > 100 µM, MIC = 128 µg/mL, and MIC = 25–100 µM, respectively). The interaction maps of Lt-MAP3 and LS-AMP-E1 are characterized by sparse contact networks dominated almost exclusively by hydrophobic interactions, with a notable absence or near-absence of stabilizing hydrogen bonds, a profile insufficient to firmly anchor the peptide within the allosteric groove. Lt-MAP2 presents a particularly instructive case: despite its poor predicted affinity and low individual potency, it was identified as the most promiscuous synergistic agent in the physicochemical analysis (Section 2.4). Its weak binding to UppP therefore suggests that its primary mechanism does not involve direct enzymatic inhibition, but rather that its high amphipathicity (µH = 0.839) likely exerts a membrane-permeabilizing effect that potentiates the action of co-administered antibiotics. A similar membrane disruption-based rationale is postulated for Lt-MAP3 and LS-AMP-E1, whose high amphipathicity is considered the dominant driver of their biological activity.

## 3. Discussion

### 3.1. Principal Findings

This in silico study explored spider venom peptides as potential allosteric inhibitors of *A. baumannii* UppP, revealing a diverse spectrum of predicted binding affinities. HADDOCK scores varied considerably across the cohort, ranging from very high affinity (LC-AMP-I1, −103.2) to weaker interactions (LS-AMP-E1, −56.3). This computational diversity correlates meaningfully with reported antimicrobial activity (MIC) and physicochemical properties, identifying promising candidates, namely LC-AMP-I1, Lycosin-II (−94.5), and GK37 (−93.3), that combine high potency (MIC ≤ 10 µM) with the most favorable HADDOCK scores. Structural analysis of their respective complexes reveals stable binding mediated by extensive interaction networks (Figure 5A,C,D).

### 3.2. High-Affinity Candidates and the Postulated Dual Mechanism of Action

The congruence observed in LC-AMP-I1, Lycosin-II, and GK37 linking potent biological activity with strong predicted affinity for the allosteric UppP target suggests a potential dual mechanism of action. On the one hand, their cationic and amphipathic nature, combined with their reported α-helical structure, facilitates interactions with and the subsequent disruption of the bacterial membrane, a rapid mechanism of action experimentally demonstrated for these peptide families. For Lycosin-II, Mg^2+^-competition assays confirmed that its bacteriostatic effect stems from direct binding to the electronegative bacterial surface, evidenced by a fourfold increase in MIC upon the addition of 5 mM Mg^2+^ [23]. The structurally related Lycosin-I exhibited broad-spectrum bactericidal activity at low micromolar concentrations, permeabilizing the cell membrane as evidenced by SYTOX Green uptake and scanning electron microscopy, while acting synergistically with conventional antibiotics in both in vitro and in vivo models [28]. Similarly, GK37 was recently shown to eradicate *S. aureus* within 30 min at 10× MIC through direct membrane perturbation, with SEM and TEM imaging revealing distorted and shriveled bacterial membranes [27].

On the other hand, the stable binding predicted at the allosteric site of UppP could specifically inhibit the recycling of the lipid carrier C55-P, a process essential for peptidoglycan synthesis. The indispensable role of UppP-mediated C55-PP dephosphorylation has been established across diverse bacterial species, from *E. coli* [7] to *B. subtilis* [8], where its inhibition leads to the accumulation of cell wall intermediates and subsequent bacterial lysis [13]. This dual targeting strategy, directed simultaneously at the bacterial membrane and at a critical transmembrane enzyme, represents a robust antibacterial approach potentially capable of mitigating the development of resistance, as bacteria would need to evolve multiple simultaneous adaptations to fully escape both membrane disruption and enzymatic blockade, a scenario that substantially reduces the probability of resistance emergence [29].

### 3.3. Low-Affinity Candidates and Synergistic Potential

In contrast, the Latarcin-3a analogs Lt-MAP2 (HADDOCK: −62.6) and Lt-MAP3 (−57.6) exhibited considerably lower predicted binding affinities, displaying interaction profiles dominated by hydrophobic contacts and a notable scarcity of hydrogen bonding. This structural profile is consistent with their weak reported intrinsic antimicrobial activity (MIC ≥ 128 µg/mL) as documented by De Moraes et al., 2022 [26]. However, prior physicochemical analysis highlighted Lt-MAP2 as the candidate with the highest predicted synergistic potential. This finding, coupled with experimental evidence from other spider-derived AMPs within this cohort, suggests that pronounced amphipathicity can drive synergy even in the absence of high intrinsic potency.

**Figure 6:**
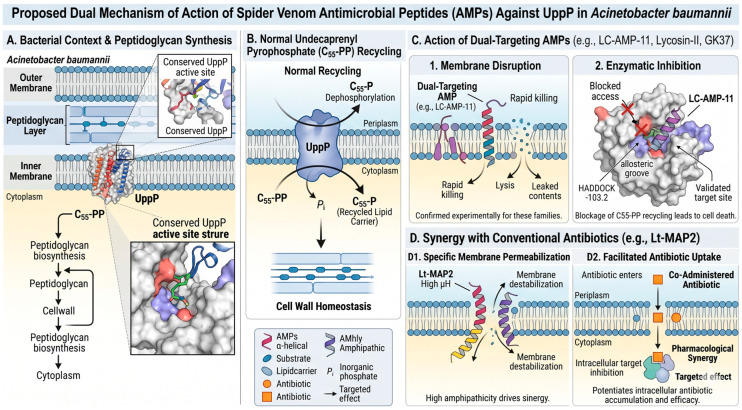
Integrative proposed mechanism of action for spider venom AMPs against *Acinetobacter baumannii* and the essential enzyme UppP. (**A**) Structural context of UppP localized in the bacterial inner membrane; the protein surface is colored by electrostatic potential (blue: positive; red: negative), highlighting the conserved catalytic regions. (**B**) Normal physiological recycling of the lipid carrier undecaprenyl pyrophosphate (blue spheres, C55-PP) to C55-P (light-blue hexagons) mediated by UppP, essential for peptidoglycan biosynthesis. (**C**) Postulated dual mechanism for high-affinity candidates (e.g., LC-AMP-I1, Lycosin-II, GK37): (1) rapid bacterial killing through direct membrane disruption by α-helical AMPs (purple cylinders); and (2) specific enzymatic blockade via allosteric inhibition of UppP. (**D**) Synergistic mechanism for high-amphipathicity candidates (e.g., Lt-MAP2): (D1) membrane permeabilization driven by high hydrophobic moment (indicated by the yellow/purple color distribution in the helix) and (D2) facilitated intracellular uptake of co-administered conventional antibiotics (orange squares), enhancing efficacy.Source: own elaboration with NanoBanana Version PRO.

As illustrated in the integrative proposed mechanism (Figure 6), these low-affinity candidates appear to operate through a distinct synergistic pathway (Figure 6D) compared to the dual-action leads. While high-affinity peptides like LC-AMP-I1 engage in both membrane disruption and direct enzymatic inhibition (Figure 6C), peptides such as Lt-MAP2 likely exert their primary effect through specific membrane permeabilization (Figure 6D(1)). This process is driven by their high hydrophobic moment (μH = 0.839), which allows the peptide to destabilize the lipid bilayer without necessarily causing immediate lysis. As shown in the model, this permeabilization facilitates the intracellular uptake of co-administered conventional antibiotics (Figure 6D(2)), enhancing their pharmacological efficacy by allowing them to reach their internal targets more effectively, a strategy particularly relevant for overcoming efflux or permeability-based resistance in *A. baumannii* [20].

Specifically, Tan et al., 2022 [22] demonstrated that LS-AMP-E1 and LS-AMP-F1 exerted synergistic or additive effects when combined with azithromycin, erythromycin, and doxycycline against both standard strains and clinical MDR isolates, reducing antibiotic MICs by up to eightfold at sub-inhibitory peptide concentrations. Similarly, Wang et al., 2014 [30] reported that Lycosin-I, a homolog of Lycosin-II in the present study, exhibited potent synergistic bactericidal activity with conventional antibiotics against MDR *A. baumannii* in a mouse thigh infection model. These observations align with the general mechanistic framework for AMP–antibiotic synergy, in which cationic amphipathic peptides increase outer-membrane permeability, thereby facilitating the intracellular accumulation of co-administered drugs [20].

Consequently, the high amphipathicity of Lt-MAP2 (μH = 0.839), while insufficient for potent independent bactericidal action, appears ideally suited for membrane permeabilization. The weak predicted interaction with UppP further supports the hypothesis that its primary therapeutic value lies in pharmacological synergy rather than direct enzymatic inhibition.

### 3.4. Binding Mode Comparison with the Control Ligand

The predicted binding modes of the peptide candidates differ substantially from that observed for the control ligand (Compound **4**), a known inhibitor of the homologous BacA enzyme. Whereas the small molecule docks into a well-defined allosteric pocket through specific interactions (Figure 4), the peptides, by virtue of their larger size and helical architecture, engage a more extensive surface area within the transmembrane region of UppP. Although less geometrically constrained than pocket occupancy, such surface interactions can be highly stable when they involve multiple contact points, as evidenced by the high-affinity candidates. This illustrates the mechanistic versatility of allosteric inhibition, which may be mediated not only through binding to discrete pockets, but also through broader surface interactions capable of inducing functionally relevant conformational changes.

### 3.5. Structure–Function Relationships

The in silico binding affinities appear to be substantially influenced by the structural properties of the individual peptides. The majority of the candidates adopt an amphipathic α-helical conformation, and both the stability of this helix and the spatial distribution of hydrophobic and cationic residues are key determinants of their interactions with membranes and transmembrane proteins. Peptides with low predicted affinity, such as Lt-MAP3 and LS-AMP-E1, may harbor less stable helices or a suboptimal residue arrangement for allosteric engagement. The literature indicates that the regularity of the amphipathic helix and a clear segregation of hydrophobic and polar faces, as observed in LS-AMP-F1, are important contributors to activity. The complete absence of reported activity for LS-AMP-G1 may be explained by the presence of proline, a residue well-known for disrupting helical secondary structure [19,31].

### 3.6. Implications, Limitations, and Future Perspectives

This computational analysis positions LC-AMP-I1, Lycosin-II, and GK37 as the most compelling candidates for dual inhibition of *A. baumannii*, combining membrane-active mechanisms with potential allosteric blockade of UppP, while Ltc-3a and LS-AMP-F1 present interesting secondary profiles and Lt-MAP2 stands out for its predicted synergistic potential. Collectively, these findings underscore the mechanistic diversity of spider venom peptides and the utility of in silico approaches for prioritizing candidates against MDR pathogens. However, several limitations inherent to the computational nature of this study must be acknowledged: all results derive exclusively from in silico analyses with no experimental validation; the UppP model was generated with AlphaFold2 in the absence of a resolved crystal structure; the docking protocol does not fully capture peptide conformational flexibility or receptor-induced-fit dynamics; and the biological complexity of the bacterial membrane environment, peptide proteolytic stability, cytotoxicity toward mammalian cells, and pharmacokinetic properties remain unaddressed. These constraints notwithstanding, the integrative framework presented here provides a rigorous and systematic basis for candidate prioritization, and experimental validation through enzymatic inhibition assays, membrane permeabilization studies, and in vivo efficacy models will be essential to confirm the therapeutic potential of these peptides.

## 4. Conclusions

This in silico study identified and structurally characterized the interaction of eight spider venom AMPs with a validated allosteric site on UppP, a conserved transmembrane enzyme essential for peptidoglycan biosynthesis in *A. baumannii* and across diverse bacterial lineages. Despite its biological importance, UppP remains pharmacologically underexploited beyond the indirect sequestering action of bacitracin. Molecular docking results revealed that LC-AMP-I1, Lycosin-II, and GK37 exhibit the most favorable HADDOCK scores (−103.2, −94.5, and −93.3, respectively), stabilized by extensive networks of hydrophobic contacts and hydrogen bonds within the allosteric groove. The alignment between these high predicted affinities and their potent reported antimicrobial activity (MIC ≤ 10 µM) supports the postulation of a dual mechanism of action combining membrane disruption with specific enzymatic blockade.

Conversely, peptides with lower binding affinity, notably Lt-MAP2, appear to exert their primary effect through membrane permeabilization that potentiates synergism with conventional antibiotics, representing a complementary therapeutic strategy. Although these findings are exclusively computational and require experimental validation through enzymatic inhibition assays, membrane permeabilization studies, and in vivo efficacy models, the integrative framework presented here establishes UppP as a viable pharmacological target for peptide-based inhibitors. Consequently, these results support the further exploration of spider venom peptides as a promising reservoir for the development of novel antibacterial agents against multidrug-resistant *A. baumannii*.

## 5. Materials and Methods

The methodological strategy employed in this study combines a systematic literature review with a robust in silico computational approach to identify and characterize spider venom-derived peptides as potential inhibitors of *A. baumannii* UppP. The workflow encompasses systematic searching and selection of candidate peptides, their in silico physicochemical characterization, detailed preparation of the molecular structures of the receptor and ligands, virtual screening via molecular docking using multiple tools, and structural validation.

### 5.1. Systematic Literature Review

A comprehensive systematic literature review was conducted to identify spider venom-derived peptides with reported antimicrobial activity and available primary sequences.

#### 5.1.1. Data Sources and Search Strategy

Systematic searches were performed across five electronic databases—PubMed, Scopus, Web of Science, EMBASE, and Google Scholar—covering publications up to 15 March 2024. A consistent search algorithm was applied, adapted to the syntax of each database, combining MeSH terms (where applicable) and keywords related to spider venom, peptides, and antibacterial activity, using Boolean operators (AND, OR). The base search string was:

(“Spider Venoms” OR “Spider Bites” OR spider* OR arachnid* OR tarantula*) AND (“Antimicrobial Cationic Peptides” OR “Defensins” OR peptide* OR toxin* OR AMP) AND (“Anti-Bacterial Agents” OR antibacterial* OR antimicrobial* OR bactericidal* OR bacteriostatic* OR “Minimum Inhibitory Concentration”)

No initial restrictions were applied regarding language or publication date. Rayyan was used for title and abstract screening. The flow diagram was created with the online R package PRISMA2020 [32] (https://estech.shinyapps.io/prisma_flowdiagram/, accessed on 10 July 2025).

#### 5.1.2. Eligibility Criteria

The following predefined criteria were applied for study selection:

Inclusion criteria: Original studies describing the isolation, synthesis, or characterization of spider venom peptides; explicit reporting of antibacterial activity; complete primary sequence available.

Exclusion criteria: Reviews, editorials, and non-original studies; peptides not derived from spider venom; absence of reported antibacterial activity; unavailable sequence; duplicates.

#### 5.1.3. Selection Process and Data Extraction

Search results were managed with Zotero (v.6.0) for duplicate removal. Two independent reviewers (Y.L., A.G.) screened titles/abstracts and full texts, resolving discrepancies by consensus or with a third reviewer (M.R.). Key information was extracted, including peptide name, sequence, source (species/family), structure (if known), reported activities (general and against *A. baumannii*), and reference, into a standardized spreadsheet (Microsoft Excel v.16.0).

### 5.2. Physicochemical Characterization and In Silico Activity Prediction

Selected peptides were characterized in silico to determine their physicochemical properties:

General properties: Length, molecular weight, isoelectric point (pI), and net charge (at pH 7.4) were calculated using PepCalc (https://pepcalc.com/, accessed on 20 October 2025) and ExPASy ProtParam (https://web.expasy.org/protparam/, accessed on 20 October 2025).

Hydrophobicity and structure: Aliphatic index and GRAVY were obtained with ProtParam. Mean hydrophobicity (H) and hydrophobic moment (µH), assuming an α-helical conformation where plausible, were calculated with HeliQuest (http://heliquest.ipmc.cnrs.fr/, accessed on 20 October 2025) using the Eisenberg hydrophobicity scale.

Antibacterial activity prediction: Artificial intelligence-based models, including DBAASP (https://dbaasp.org/tools?page=linear-amp-prediction, accessed on 21 October 2025), were used to predict the antibacterial potential of the identified peptides. Synergy predictions against *A. baumannii* ATCC 19606 were also generated using the DBAASP synergy prediction tool (https://dbaasp.org/tools?page=synergy-prediction, accessed on 21 October 2025), serving as an additional prioritization criterion.

Physicochemical data were compiled in a comparative table. For heatmap-based visualization of physicochemical properties, each variable was standardized by Z-score normalization, defined as Z = (X − μ)/σ, where X is the observed value, μ is the mean of the property across all peptides, and σ is its standard deviation. This standardization enabled the comparison of properties measured on different scales by transforming all variables to a distribution with mean 0 and standard deviation 1. Python (v.3.9) libraries including Matplotlib (v.3.9.4) and Seaborn (v.0.13.2) were used to generate descriptive plots of these properties (e.g., distributions of length, charge, and hydrophobicity).

### 5.3. Molecular Structure Preparation

#### 5.3.1. Receptor: *A. baumannii* UppP

The amino acid sequence of *A. baumannii* UppP (strain ACICU) was retrieved in FASTA format from UniProt (ID: B2HXY4). This strain was selected because it is a thoroughly characterized MDR clinical isolate representative of globally prevalent *A. baumannii* lineages (International Clone II), thereby conferring greater clinical relevance to the findings.

A 3D structural model was generated from this sequence using AlphaFold2 via the AlphaFold Database (https://alphafold.ebi.ac.uk/, accessed on 19 March 2025). Model quality was assessed using pLDDT confidence scores, ProSA-web (https://prosa.services.came.sbg.ac.at/prosa.php, accessed on 25 November 2025), and MolProbity (http://molprobity.biochem.duke.edu/, accessed on 25 November 2025) [33].

#### 5.3.2. Ligands: Spider Peptides and Control Compound

Three-dimensional structures of the selected peptides were prepared. For those lacking experimental structures, the PEP-FOLD 3.5 server was employed (https://mobyle.rpbs.univ-paris-diderot.fr/cgi-bin/portal.py#forms::PEP-FOLD3, accessed on 21 October 2025) [34]. The generated peptide structures were subsequently validated for stereochemical and energetic quality using ProSA-web [35] and MolProbity [33].

For the selection of the positive control, given that neither the literature nor databases such as DrugBank (https://go.drugbank.com/, accessed on 21 October 2025) report established inhibitors for the *A. baumannii* UppP target structure, a literature search was conducted for inhibitors of homologous enzymes. The positive control selected was Compound 4—5-((3-fluoro-4-(pyrrolidin-1-yl)phenyl)sulfonamido)thiophene-2-carboxylic acid—which has demonstrated confirmed activity against BacA (a functional homolog of UppP), as reported by Jukič et al. (2022) [6]. The structure of this ligand was derived from its SMILES string ([H]OC(=O)C1=C([H])SC(=C1[H])S(=O)(=O)N([H])C1=C([H])C([H])=C(N2C([H])([H])C([H])([H])C([H])([H])C2([H])[H])C(F)=C1[H]), retrieved from the RCSB Chemical Sketch tool (https://www.rcsb.org/chemical-sketch, accessed on 21 October 2025). Its 3D structure was generated by converting the SMILES string to PDB format using the NovoPro online converter (https://www.novoprolabs.com/tools/smiles2pdb, accessed on 21 October 2025).

### 5.4. Molecular Docking Protocol

Binding site definition: Protein–peptide docking was performed to predict binding modes and evaluate the affinity of candidate peptides. The binding site was defined based on the conserved catalytic region of UppP, homologous to that reported by Jukič et al. (2022) [6], which identified the key active-site residues: Glu17, Glu21, Ser27, Arg174, and Ser175, corresponding to the conserved BacA motifs BacA1 (residues 16–35) and BacA2 (residues 151–180). These catalytic residues were designated as active residues in HADDOCK to direct docking toward the functionally relevant region of the UppP receptor.

Docking procedure: HADDOCK 2.4 (High Ambiguity Driven protein–protein DOCKing) (https://www.bonvinlab.org/software/haddock2.4/, accessed on 23 October 2025) [36,37] was used to perform peptide–protein docking. HADDOCK was selected over other docking programs due to its unique capacity to integrate experimental or bioinformatic data through Ambiguous Interaction Restraints (AIRs), its specialization in protein–peptide complexes, and its data-driven docking approach that allows incorporation of prior knowledge of binding sites.

A more negative score indicates greater binding affinity and higher complex quality. The best models (clusters) were selected based on: (1) the lowest (most negative) HADDOCK score; (2) the smallest intra-cluster RMSD (greater structural consistency); (3) favorable interaction energies. As a general reference, scores below −100 are indicative of high-affinity complexes, though this threshold may vary depending on the system. Visual and interaction analysis, including hydrogen bonds and hydrophobic contacts, was performed using PyMOL (v.2.5, The PyMOL Molecular Graphics System, Schrödinger, LLC, New York, NY, USA) and Discovery Studio Visualizer (v.21.1, Dassault Systèmes BIOVIA, 2020).

### 5.5. Software and Resources

The following tools and resources were used throughout the study: PubMed, Scopus, Web of Science, EMBASE, and Google Scholar (last accessed April 2026). Reference management was performed using Zotero v.6.0, and data tabulation with Microsoft Excel v.16.0. Protein information and structures were retrieved from UniProtKB (Release 2026_01), AlphaFold DB (v.2025_03), and PDB. Structure validation was conducted via ProSA-web, MolProbity v.4.5.2, and ExPASy ProtParam. Physicochemical properties were analyzed using PepCalc, HeliQuest, iAMPpred, and AMPScanner v.2. Peptide modeling was performed with PEP-FOLD3, while docking simulations were executed using DrugBank v.5.1.12, CB-Dock2, AutoDock Tools v.1.5.7, AutoDock Vina v.1.2.3, and HawkDock V2. Molecular visualizations were generated using PyMOL v.2.5 and Discovery Studio Visualizer v.21.1.

## Figures and Tables

**Table 1:** Spider venom peptides with reported activity against *A. baumannii* selected from the literature.

Peptide Name	Author (Year)	Origin (Species)	Family	Sequence	Structure	MIC vs. *A. baumannii*
LC-AMP-I1	Wang et al. (2025) [21]	*Lycosa coelestis*	Lycosin	GRMQEFIKKLKAYLRKMKEKFSQIS	alpha-helical, amphipathic	5–10 µM
Ltc-3a	De Moraes et al. (2022) [26]	*Lachesana tarabaevi*	Latarcin	SWKSMAKKLKEYMEKLKQRA	alpha-helical	4 µg/mL
Lt-MAP2	De Moraes et al. (2022) [26]	Synthetic Analog	Latarcin	LIKKLKEYLKKLI	alpha-helical	8 µg/mL
Lt-MAP3	De Moraes et al. (2022) [26]	Synthetic Analog	Latarcin	LAKKLAKYLKKAL	alpha-helical	128 µg/mL
LS-AMP-E1	Tan et al. (2022) [22]	*Lycosa sinensis*	Lycosin	AGMKNIIDAIKKKLGGKL	alpha-helical, amphipathic	25–100 µM (vs. MDR)
LS-AMP-F1	Tan et al. (2022) [22]	*Lycosa sinensis*	Lycosin	TGLGKIGYLMKKLLSKAKV	alpha-helical, amphipathic	3.1–25 µM (vs. MDR)
Lycosin-II	Wang et al. (2016) [23]	*Lycosa singoriensis*	Lycosin	VWLSALKFIGKHLAKHQLSKL	alpha-helical, amphipathic	3.1–6.3 µM
GK37	Wang et al. (2024) [27]	*Oxyopes forcipiformis*	Oxyopidae	GKFSIFGKILSSIAKVFKGVGKVRKSFQNARDLDKPKNRE	alpha-helical, amphipathic	6.209 µM

MIC: Minimum Inhibitory Concentration; MDR: Multidrug-Resistant.

**Table 2:** Physicochemical properties and in silico predicted antimicrobial activity.

Peptide Name	Length (aa)	Net Charge	Aliphatic Index	Grand Average of Hydropathicity (GRAVY)	Hydrophobicity (H)	Hydrophobic Moment (µH)	DBAASP Antimicrobial Prediction
LC-AMP-I1	25	6	66.4	−0.876	0.182	0.573	Antimicrobial
Ltc-3a	20	5	49	−1.360	0.058	0.575	Antimicrobial
Lt-MAP2	13	4	180	−0.008	0.444	0.839	Antimicrobial
Lt-MAP3	13	5	143	−0.015	0.288	0.701	Antimicrobial
LS-AMP-E1	18	4	119	−0.061	0.241	0.468	Antimicrobial
LS-AMP-F1	19	5	123	0.216	0.399	0.352	Antimicrobial
Lycosin-II	21	4.2	135	0.271	0.58	0.26	Antimicrobial
GK37	40	8	76	−0.59	0.156	0.259	Antimicrobial

DBAASP: Database of Antimicrobial Activity and Structure of Peptides. Hydrophobicity (H) and hydrophobic moment (µH) were calculated assuming an α-helical structure.

## Data Availability

Data are contained within the article.
